# Delta-like 4/Notch signaling promotes *Apc*^*Min/*+^ tumor initiation through angiogenic and non-angiogenic related mechanisms

**DOI:** 10.1186/s12885-016-3036-0

**Published:** 2017-01-13

**Authors:** Marina Badenes, Alexandre Trindade, Hugo Pissarra, Luís Lopes-da-Costa, António Duarte

**Affiliations:** Centro Interdisciplinar de Investigação em Sanidade Animal (CIISA), University of Lisbon, Lisbon, Portugal

**Keywords:** Notch expression, Dll4, Ubiquitous knockout, Endothelial-specific knockout, Tumor stem cells, Angiogenesis, *Apc*^*Min/*+^ mouse

## Abstract

**Background:**

Delta like 4 (Dll4)/Notch signaling is a key regulator of tumor angiogenesis. Additionally, the role of Dll4 has been studied on tumor stem cells. However, as these cells are implicated in tumor angiogenesis, it is conceivable that the effect of Dll4 on these cells may be a consequence of its angiogenic function. Our aim was to evaluate the expression and dissect the functions of Dll4 in the *Apc* ^*Min/*+^ model of colorectal cancer.

**Methods:**

We evaluated the protein expression pattern of Dll4 and other Notch members in the *Apc* ^*Min/*+^ tumors relatively to the normal gut and compared endothelial-specific with ubiquitous Dll4 knockout mice on an *Apc* ^*Min/*+^ background.

**Results:**

All Notch pathway members were present in the normal small and large intestine and in the adenomas of the same regions. Dll4, all Notch receptors and Hes1 expression seemed upregulated in the tumors, with some regional differences. The same members and Hes5, instead of Hes1, presented ectopic expression in the tumor parenchyma. Dll4 expression was most pronounced in the tumor cells but it was also present in the tumor blood vessels and in other stromal cells. Ubiquitous and endothelial-specific Dll4 deletion led to an equivalent reduction of tumor growth because of a similarly marked tumoral angiogenic phenotype promoting non-productive vasculature and consequently hypoxia and apoptosis. The ubiquitous Dll4 inhibition led to a stronger decrease of tumor multiplicity than the endothelial-specific deletion by further reducing tumor proliferation and tumor stem cell density through upregulation of the cyclin-dependent kinase inhibitors 1C and 1B and downregulation of Myc, Cyclin D1 and D2 independently of β-catenin activation. This phenotype was associated to the observed increased epithelial differentiation deviated towards the secretory lineages by Atoh1 and Klf4 upregulation only in the ubiquitous Dll4 mutants.

**Conclusions:**

Dll4 seems to promote *Apc* ^*Min/*+^ tumorigenesis through both angiogenic and non-angiogenic related mechanisms.

**Electronic supplementary material:**

The online version of this article (doi:10.1186/s12885-016-3036-0) contains supplementary material, which is available to authorized users.

## Background

Colorectal cancer (CRC), one of the most frequent malignancies in the Western world, is commonly associated with mutations in the tumor suppressor *Adenomatous polyposis coli (APC)* gene both in hereditary [1] and in sporadic CRC [2]. Mutations of this gene constitutively activate β-catenin target genes causing tumorigenesis [3]. The *Apc* ^*Min/*+^ mouse, which spontaneously develops multiple intestinal neoplasms (Min) in the small and large intestine, is considered a good model to study CRC [4, 5].

Studies revealed that all of the Notch receptors, their ligands and some of their effectors (Hes1, 5, 6, 7 and Atoh1) are expressed in the normal intestinal crypts, the main niche for stem cells. Dll1 and Dll4 interact with Notch1 and 2 in the normal gut to maintain the homeostasis of intestinal stem cells [6] by repressing the cyclin-dependent kinase inhibitors *Cdkn1b and Cdkn1c* [7]. The Notch signaling pathway also promotes the enterocyte/colonocyte differentiation [8, 9], while Atoh1 and Klf4, which are transcriptionally repressed by Hes1, specify secretory (goblet, enteroendocrine, and Paneth) cell differentiation [10, 11].

Activation of Notch1 together with Wnt signalling seems to be essential to trigger CRC initiation by maintaining the self-renewal of tumor stem cells [12, 13]. These cells share some characteristics with normal stem cells but have accumulated oncogenic mutations and lost growth control. They possess the strongest tumor-initiating potential of all tumor cells and promote tumor growth and resistance to many current therapies, including chemo and radiotherapy [14]. Several intestinal stem cell markers have been investigated, such as the leucine-rich repeat-containing G-protein-coupled receptor 5 (Lgr5) and the B cell–specific Moloney murine leukemia virus insertion site 1 (Bmi1) [15–17]. These two markers are also present in the normal gut in two functionally different intestinal stem cell populations; Lgr5 is present in mitotically active stem cells that are sensitive to irradiation and Wnt modulation, while Bmi1 is a marker for a reserve population of resistant quiescent injury-inducible stem cells [18]. In CRC both Lgr5 and Bmi1 positive stem cell populations are associated with cancer initiation and progression [15, 17, 19] and are regulated by the Notch pathway [20, 21].

Accumulating evidence has shown that tumor stem cells promote tumor angiogenesis and that their maintenance depends upon functional angiogenesis [22]. It is currently widely recognized that tumor growth and maintenance is dependent on the expansion of the individual’s vasculature to the center of the tumor [23]. Previous work showed that the inhibition of Dll4/Notch represses tumor growth by promoting dysfunctional an immature tumoral angiogenesis in a variety of xenograft and autochthonous mouse models [24–27]. In xenograft models of CRC anti-Dll4 therapy seems to additionally reduce the frequency of tumor stem cells [28, 29].

Thus, we set out to characterize the expression pattern of Dll4 and other Notch members in the *Apc* ^*Min/*+^ tumors relatively to the normal intestine and compare endothelial-specific with ubiquitous *Dll4* loss-of-function mutants to address the role of Dll4 in intestinal tumor development in the *Apc* ^*Min/*+^ model. In particular, we aimed to assess whether an effect of Dll4 signalling on the *Apc* ^*Min/*+^ tumor stem cells was solely a consequence of its action on tumor angiogenesis or if other, more direct, mechanisms might be involved.

## Methods

### Experimental animals

All experiments were conducted in accordance with the Portuguese legislation for the use of animals for experimental purposes (Decreto-Lei n° 129/92 and Portaria n° 1005/92, DR n° 245, série I-B, 4930-42) and with the European Union legislation (Directive n. 86/609/EEC, from the 24^th^ November 1986). All animal-involving procedures in this work were approved by the national regulatory agency (DGAV – Direção Geral de Alimentação e Veterinária) and the Institutional Animal Care and Use Committee (CEBEA – Comissão de Ética e Bem-Estar Animal) (Approval ID: PTDC/CVT/71604/2006). All sections of this report adhere to the ARRIVE Guidelines for reporting animal research [30]. A completed ARRIVE guidelines checklist is included in Additional file 1.

Mice were maintained in a conventional facility in a 12-h light/dark cycle, in ventilated cages with corncob as bedding, and were given access to standard laboratory diet and water ad libitum. The welfare of the mice was regularly monitored.

Mutant C57BL/6J-*Apc* ^*Min/*+^/J (*Apc* ^*Min/*+^) mice were purchased from the Jackson Laboratory (Bar Harbor, ME).

Two wild type C57BL/6J male mice were used to analyse Notch pathway expression in the normal intestine and two *Apc* ^*Min/*+^ male mice were used to characterize their expression in intestinal tumors.

*Apc* ^*Min/*+^ mice were crossed with *Dll4* conditional homozygous mice (*Dll4* ^*lox/lox*^). The resulting *Apc* ^*Min/*+^; *Dll4* ^*lox/lox*^ progeny was crossed with *VE-cadherin-Cre-ERT2* mice to produce endothelial-specific inducible *Dll4* loss-of-function (endo*Dll4* ^*-/-*^) or with *Cag-Cre-ERT2* mice to produce ubiquitous inducible loss-of-function (ubiq*Dll4* ^*-/-*^). When these mice reached 6 weeks, recombinase cre activity was induced by daily i.p. tamoxifen administration (50 mg/kg in 10% ethanol plus 90% cremophor), during 5 days as in [31]. Tamoxifen treated cre-negative mice were used as controls. Twelve males per group were used in the analysis.

### Macroscopic analysis of the intestine

At 18 weeks of age the animals were sacrificed and the small and large intestine were excised, flushed and opened longitudinally. The macroscopic small and large intestinal tumors of *Apc* ^*Min/*+^ *Dll4* mutant mice and controls were counted and measured with a calliper under the dissection microscope in a blinded manner. Tumor volume was calculated assuming a hemispherical shape for the small bowel tumors and a spherical shape for large intestinal tumors. The volumes of all tumors from each mouse were added to give the overall tumor burden per animal. Normal WT small and large intestine and the tumors of these regions from *Apc* ^*Min/*+^ Dll4 mutant mice and controls were collected.

### Histopathological analysis

The collected samples were fixed in 10% formalin solution for 48 h, dehydrated in alcohol, cleared in xylene, embedded in paraffin, sectioned at 4 μm and stained with hematoxylin (Fluka AG Buchs SG Switzerland) and eosin Y (Sigma, St. Louis, MO) for histopathological analysis. The lesions observed on the H&E sections from *Apc* ^*Min/*+^ mice were classified as hyperplasias, when only an increase of the number of cells was observed, or as adenomas with low and high-grade dysplasia based on the alterations of the shape of the nucleus, the nucleus to cytoplasm ratio, cell polarity, chromatin pattern, and changes in gland architecture.

Periodic Acid-Schiff (PAS) staining (Sigma, St. Louis, MO) was used to mark the intestinal goblet cells. These cells were counted in the intestine PAS stained sections using a 400× magnification.

### Immunohistochemical analysis

Three series of sequential 4 μm sections of paraffin embedded-WT normal small and large intestine and *Apc* ^*Min/*+^ intestinal adenomas from two mice each were used (2 sections per slide).

After dewaxing and rehydration, endogenous peroxidase activity was quenched (15 min, 1% H_2_O_2_) and antigen retrieval was performed (20 min at 95 °C in 10 mmol/L sodium citrate buffer, pH 6). The primary antibodies to mark Dll1, Dll4, Notch1-3, Hes1 and 5 (Abcam, Cambridge, UK) and Jagged1, Jagged2, Dll3 and Notch4 (Santa Cruz Biothecnology, California, USA) were diluted in PBS containing 2% bovine serum albumin, and incubated overnight at 4 °C with the tissue sections. These antibodies have been previously validated [32–34]. The following morning, the tissue sections were incubated with goat anti-rabbit (Merck Millipore, Massachusetts, USA) or rabbit anti-goat (Santa Cruz Biothecnology, California, USA) horseradish peroxidase–labeled secondary antibody and the staining was revealed with ImmPACT DAB Peroxidase Substrate (100 μl, Vector Laboratories, Burlingame, USA).

The sections were examined under an Olympus Bx51 microscope with the 40×/0.75 objective (UPlanfL). The images were captured with a Olympus DP21 camera.

A semi-quantitative analysis of the protein expression in the epithelium was performed by a pathologist in a blinded manner. Twelve representative fields for each staining were evaluated according to a scoring criteria adapted from [35]. Staining intensity was scored as 0 (negative), 1 (weak), 2 (moderate), and 3 (strong). Percent positivity was converted to scores as 0 (0%), 1 (1–2%), 2 (3–15%), 3 (16–30%), 4 (31–50%), 5 (51–75%) and 6 (75–100%). A final score was obtained by multiplying the percentage score by the intensity score.

### Immunofluorescence analysis

Small and large intestinal tumors were fixed in a 4% (w/v) paraformaldehyde in PBS solution at 4 °C for 1 h, cryoprotected in 15% (w/v) sucrose in PBS solution, embedded in 7.5% (w/v) gelatin in PBS solution, snap frozen in liquid nitrogen and cryosectioned in 10 and 20 μm-thick sections. The following primary antibodies were used: anti-PECAM-1, anti-E-cadherin (BD Biosciences, San Jose, USA), anti-α-SMA, anti-PCNA, anti-Lgr5, anti-HIF1α, anti-Cyclin D1 (Abcam, Cambridge, UK), anti-Dll4 (R&D Systems, Minneapolis, USA), anti-lysozyme (Dako, Glostrup, Denmark), anti-non-phospho (active) β-catenin (Cell Signaling Technology, Danvers, USA). Species-specific secondary antibodies conjugated with Alexa Fluor 488 and 594 (Invitrogen, Carlsbad, CA) were used to reveal primary antibody binding. Tissue sections were incubated with primary antibody overnight at 4 °C and with secondary antibody for 1 h at room temperature. Nuclei were counterstained with 4′, 6-diamidino-2-phenylindole dihydrochloride hydrate (DAPI; Molecular Probes, Eugene, OR).

Fluorescent immunostained sections were examined under a Leica DMRA2 fluorescence microscope with a Leica HC PL Fluotar 10 and 20×/0.5 NA dry objective, captured using Photometrics CoolSNAP HQ, (Photometrics, Friedland, Denmark), and processed with Metamorph 4.6-5 (Molecular Devices, Sunnyvale, CA, US). Morphometric analyses were performed using the NIH ImageJ 1.37v program. After transforming the RGB images into binary files, the percentage of white pixels per field was defined as a positive signal.

Under the effect of 2-2-2 tribromoethanol anaesthesia, biotin-conjugated lectin from *Lycopersicon esculentum* (100 μg/100 μl of PBS) or 1% Evans’ Blue solution (Sigma, St. Luis, MO, US) were administered in the caudal vein to mark vessel perfusion and extravasation, respectively. Both solutions were allowed to circulate for 5 min before the vasculature was transcardially perfused with 4% (w/v) paraformaldehyde in PBS solution for 3 min. Tumor samples were collected and processed as described above. Tissue sections were stained and tumor perfusion was quantified by determining the percentage of red PECAM-positive structures that were co-localized with Streptavidin-Alexa 488 (Invitrogen, Carlsbad, CA, US) signals. Evans’ Blue is red fluorescent and extravasation was visualized in contrast to green fluorescent vascular structures.

Apoptosis was measured using the TUNEL assay (Roche, Mannheim, Germany).

### Quantitative transcriptional analysis

Intestinal tumors were snap frozen in liquid nitrogen until RNA extraction (Qiagen RNeasy). Using the SuperScript® III First-Strand Synthesis SuperMix for qRT-PCR (Invitrogen, Carlsbad, CA, USA), first-strand cDNA was synthesized from total RNA. Real-time PCR analysis was performed using the comparative C_T_ method [36]. Primer pair sequences are listed in Additional file 2: Table S1. Gene expression was normalized to *β-actin* and in the case of genes expressed in the vasculature it was additionally normalized to *Pecam-1*.

### Statistical analysis

To compare measurements between control and test groups, the Mann–Whitney-Wilcoxon test was performed using the Statistical Package for the Social Sciences v15.0 (Chicago, IL). Results are presented as relative average ± SEM. *P*-values <0.05 and <0.01 were considered significant (*) and highly significant (**), respectively.

## Results

### Notch pathway is upregulated in *Apc*^*Min/*+^ small and large intestine tumors relatively to the normal intestine

Previous RNA-based studies described the expression pattern of the Notch pathway components in the normal gut [37, 38]. However, there is still limited information about its expression in the *Apc* ^*Min/*+^ mouse intestinal tumors. The only existing studies in the *Apc* ^*Min/*+^ adenomas indicated that the expression of Notch receptors and ligands was similar to that observed in the crypts, Hes1 was detected uniformly [8] and Jagged1 was overexpressed in the tumor tissue with concomitant Notch1 and 2 activation [39]. A more complete overview has not been produced. Therefore we evaluated the protein expression pattern of the ligands (Dll1, 3 and 4 and Jagged1 and 2), receptors (Notch1-4) and chosen effectors (Hes1 and 5) in the normal WT gut. We analysed the presence of these components in the crypts and villi of the small intestine, in the bottom and top of the crypts of the large intestine and in the *lamina propria* of both regions as summarized in Additional file 3: Table S2. Then we evaluated the expression pattern of Notch components in the *Apc* ^*Min/*+^ mouse small and large intestinal adenomas and compared it with that of normal WT gut epithelium.

**Fig. 1:**
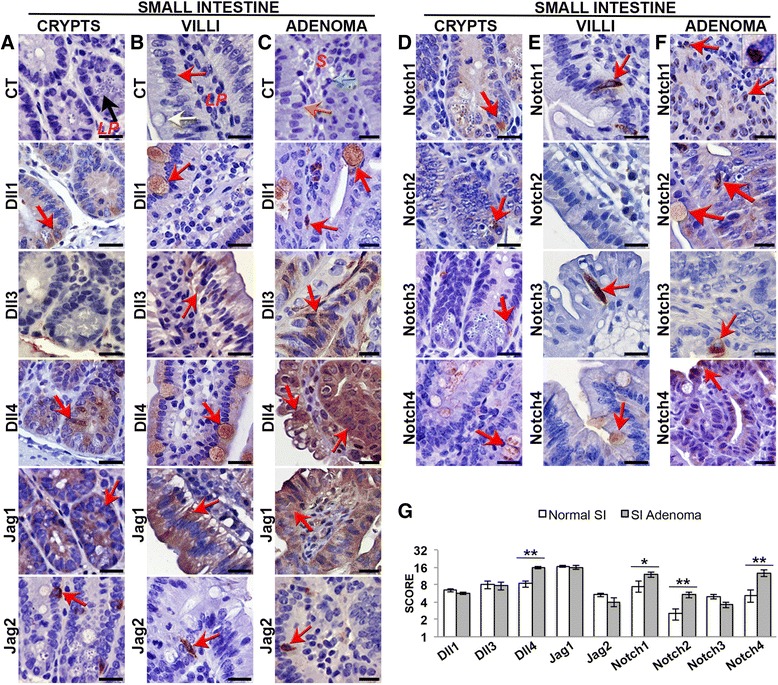
Notch pathway expression pattern in the small intestine and in *Apc* ^*Min/*+^ small intestinal adenomas. **a**-**g** Immunohistochemical analysis of Notch ligands and receptors expression (indicated by a *red arrow*) in three series of sequential sections of the normal small intestine of C57Bl/6 mice, and in *Apc* ^*Min/*+^ small intestinal adenomas (*n* = 2 per group, and 2 sections per slide). Control staining was performed with the specific species IgG. Notch ligands (**a**-**c**) and receptors (**d**-**f**) expression in the small intestinal crypts (**a**, **d**), villi (**b**, **e**) and adenomas (**c**, **f**). Scale bar = 20 μm. *Black arrow* indicates a Paneth cell (**a**). *Red arrow* points to an enterocyte (**b**); *Yellow arrow* indicates a goblet cell (**b**); *Orange arrow* in (**c**) indicates the tumor epithelium; *Green arrow* points out a neutrophil (**c**). *LP* lamina propria, *S* stroma. **g** Graphic bars represent the average staining score ± SEM for each protein in the normal small intestine (SI) and adenomas of the same region. *P < 0.05; **P < 0.01

In the normal small intestinal crypt epithelium our protein expression analysis revealed that all Notch members, except Dll3, were present (Figs. 1a,d and 3a, d). Most pathway members were expressed at the bottom of the crypts, where the proliferative and Paneth cells are located (Figs. 1a, d and 3a, d). Jagged1, however, was expressed throughout the crypts, but absent in Paneth cells (Fig. 1a), whereas Notch4 was only present in goblet cells (Fig. 1d).

In the normal small intestinal villi epithelium, Dll1, Dll4 (Fig. 1b) and seldom Notch4 (Fig. 1e) were expressed in differentiated goblet cells. Dll3, Jagged1 and 2 (Fig. 1b), Notch1 and 3 (Fig. 1e) and Hes1 and 5 (Fig. 3b and e) were found in enterocytes. Notch2 seemed absent in this region (Fig. 1e).

**Fig. 2:**
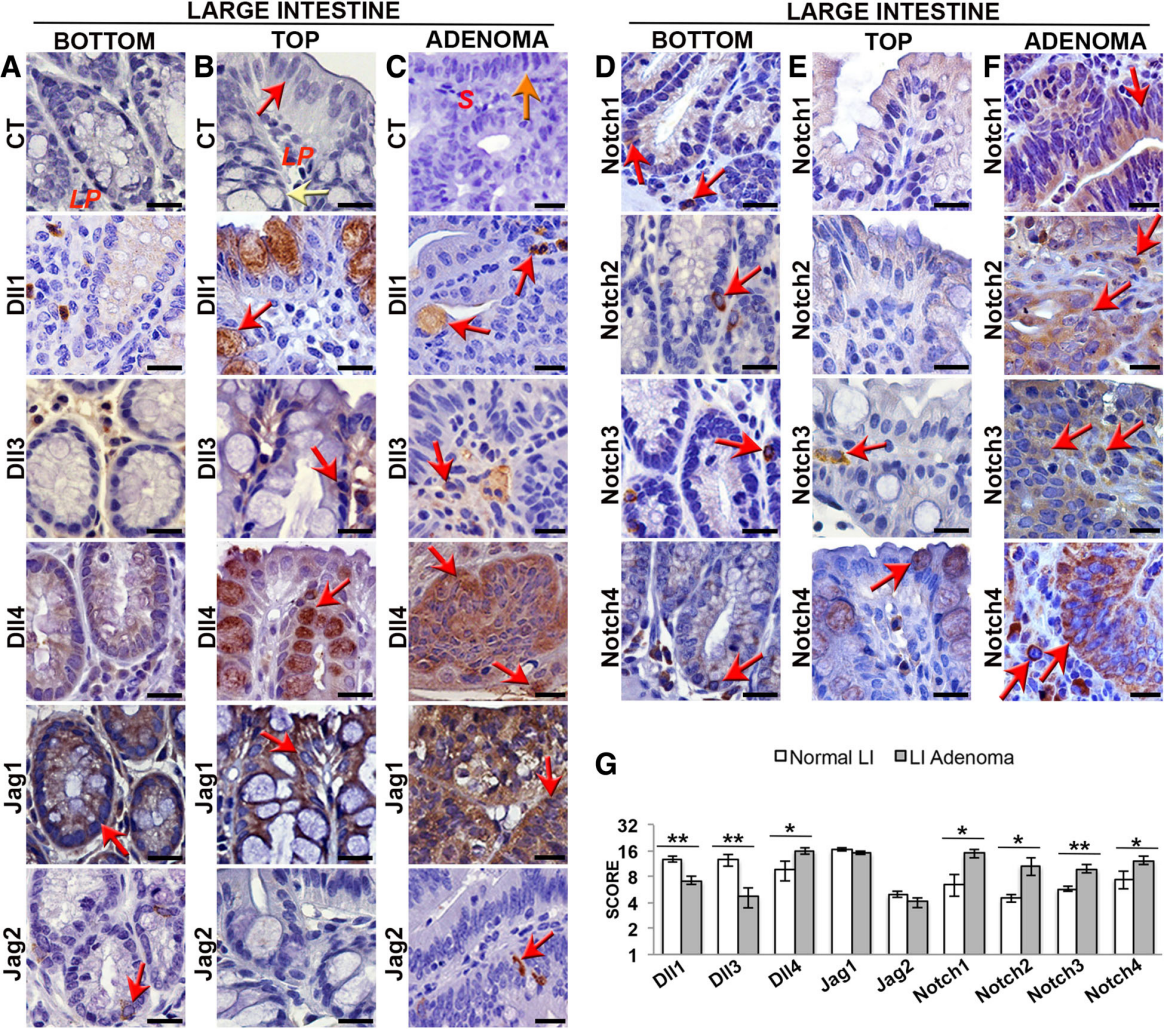
Notch pathway expression pattern in the large intestine and in *Apc* ^*Min/*+^ large intestinal adenomas. **a**-**g** Immunohistochemical analysis of Notch ligands and receptors expression (indicated by a *red arrow*) in three series of sequential sections of the normal large intestine of C57Bl/6 mice, and in *Apc* ^*Min/*+^ large intestinal adenomas (*n* = 2 per group, and 2 sections per slide). Control staining was performed with the specific species IgG. Notch ligands (**a**-**c**) and receptors (**d**-**f**) expression in the large intestine in the bottom (**a**, **d**) and top (**b**, **e**) of the crypts and in adenomas (**c**, **f**). Scale bar = 20 μm. *Red arrow* points to a colonocyte (**b**); *Yellow arrow* indicates a goblet cell (**b**); *Orange arrow* in (**c**) indicates the tumor epithelium; *LP* lamina propria, *S* stroma. **g** Graphic bars represent the average staining score ± SEM for each protein in the normal large intestine (LI) and adenomas of the same region. *P < 0.05; **P < 0.01

**Fig. 3:**
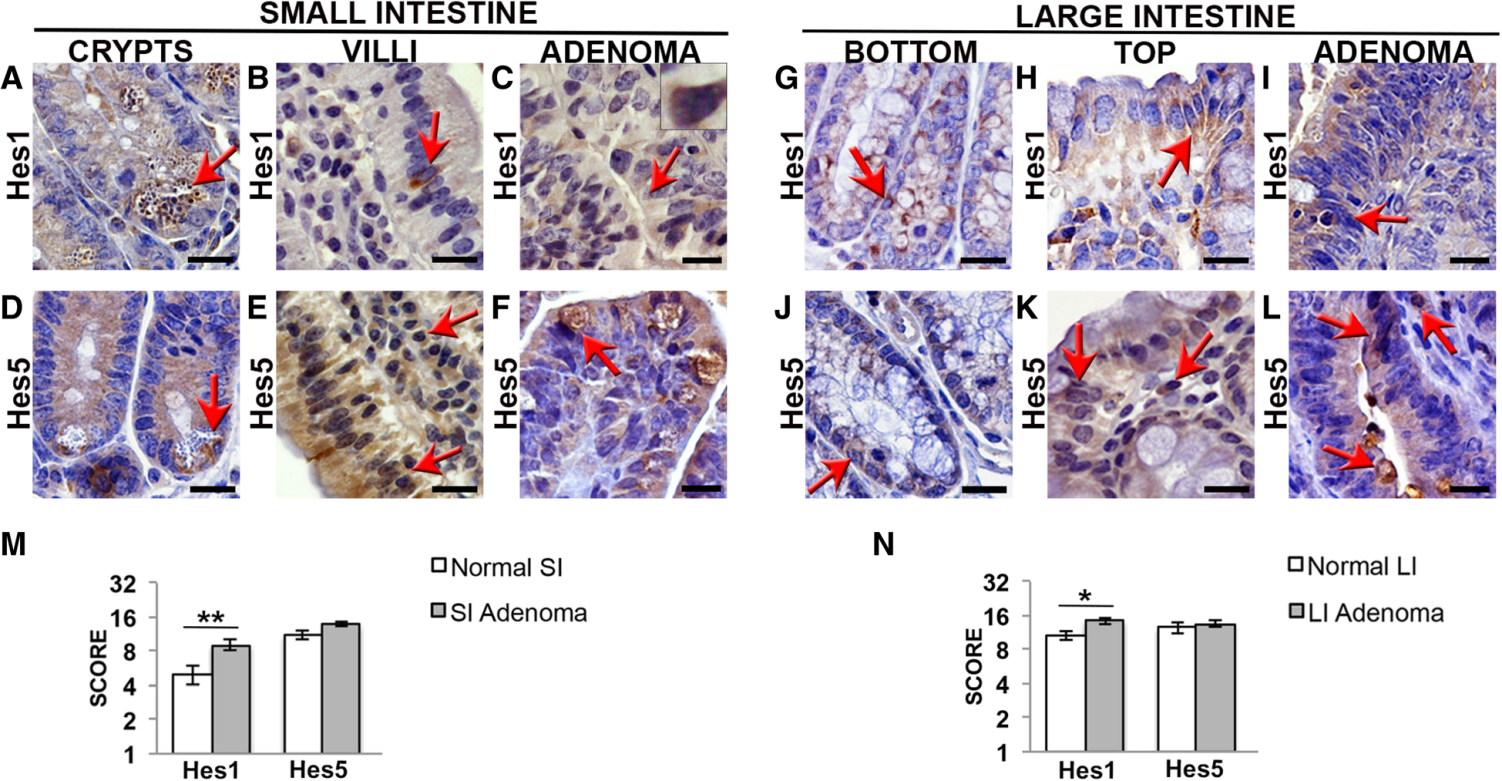
Hes1 and Hes5 expression pattern in the small and large intestine and in *Apc* ^*Min/*+^ intestinal adenomas. **a**-**l** Immunohistochemical analysis of Notch effectors Hes1 and Hes5 expression (indicated by an *arrow*) in three series of sequential sections of the normal small and large intestine of C57BL/6 mice, and in *Apc* ^*Min/*+^ small and large intestinal adenomas (*n* = 2 per group, and 2 sections per slide). Hes1 (**a**-**c**) and Hes5 (**d**-**f**) expression in the small intestinal crypts (**a**, **d**), villi (**b**, **e**) and adenomas (**c**, **f**). Hes1 (**g**-**i**) and Hes5 (**j**-**l**) expression in the large intestine in the bottom (**g**, **j**) and top (**h**, **k**) of the crypts and in adenomas (**i**, **l**). *Scale bar* = 20 μm. **m**-**n** Graphic bars represent the average staining score ± SEM for each protein in the normal tissue and adenomas of the small intestine (SI) (**m**) and large intestine (LI) (**n**). *P < 0.05; **P < 0.01

In the bottom of the crypts of the normal large intestine, Jagged1 (Fig. 2a) and Hes5 (Fig. 3j) were diffusely expressed in the epithelium. Jagged2 (Fig. 2a), Notch1-4 (Fig. 2d) and Hes1 (Fig. 3g) were found in scattered cells within the lower part of the crypts. Dll1, 3 and 4 seemed mostly absent in the epithelium of this region (Fig. 2a).

In the top of the crypts of the normal large intestine, Dll1, Dll4 (Fig. 2b) and Notch4 (Fig. 2e) were expressed in goblet cells. Dll3, Jagged1 (Fig. 2b), Hes5 and Hes1 (Fig. 3h and k) were expressed in colonocytes. Jagged2 (Fig. 2b), Notch1, 2 and 3 (Fig. 2e) seemed absent in the epithelium of this region.

All of the members were also present in some cells in the small and large intestinal *lamina propria*, mainly Hes5 (Fig. 3e and k) and Dll3 in the large intestine (Fig. 2a and b).

In the *Apc* ^*Min/*+^ intestinal adenomas, the Notch pathway members were mostly expressed in some stromal cell populations, such as neutrophils, and in tumor cells, predominantly at the periphery of the tumor mass (Figs. 1c, f, 2c, f and 3c, f, i, l).

The expression level of Dll1, Dll3, Jagged1, Jagged2 (Fig. 1a-c and g), Notch3 (Fig. 1d-f and g), and Hes5 (Fig. 3d-f and m) was similar in the small intestine adenomas and the normal tissue epithelium. Dll4 (Fig. 1a-c and g), Notch1, 2 and 4 (Fig. 1d-f and g), and Hes1 (Fig. 3a-c and m) appeared upregulated.

We observed ectopic expression of Dll4 (Fig. 1c), Notch2 and 4 (Fig. 1f) and Hes5 (Fig. 3f) in the small intestine tumor epithelium. Dll4 also appeared in other tumor cells, besides goblet cells (Fig. 1c). Notch2 (Fig. 1f) and Hes5 (Fig. 3f) were present in the goblet cells. Notch4 was not detected in the goblet cells, being present in other epithelial cell types (Fig. 1f).

In the epithelium of the large intestine adenomas, Jagged1 and Jagged2 had a similar expression level to the normal tissue (Fig. 2a-c and g). Dll1 and Dll3 seemed less expressed in the tumor epithelium (Fig. 2a-c and g), and Dll4 (Fig. 2a-c and g), all Notch receptors (Fig. 2d-g), and Hes1 (Fig. 3g-i and n) seemed upregulated. We observed ectopic expression of Dll4 (Fig. 2c), Notch4 (Fig. 2f), and Hes5 in the tumor epithelium (Fig. 3l), which was similar to that described above for the small intestinal adenomas. In this region Notch1 and Notch3 was also ectopically expressed, appearing diffusely in the tumor epithelium (Fig. 2f).

### Endothelial-specific but mainly ubiquitous inhibition of Dll4 function delays the development of intestinal tumors in *Apc*^*Min/*+^ mouse

**Fig. 4:**
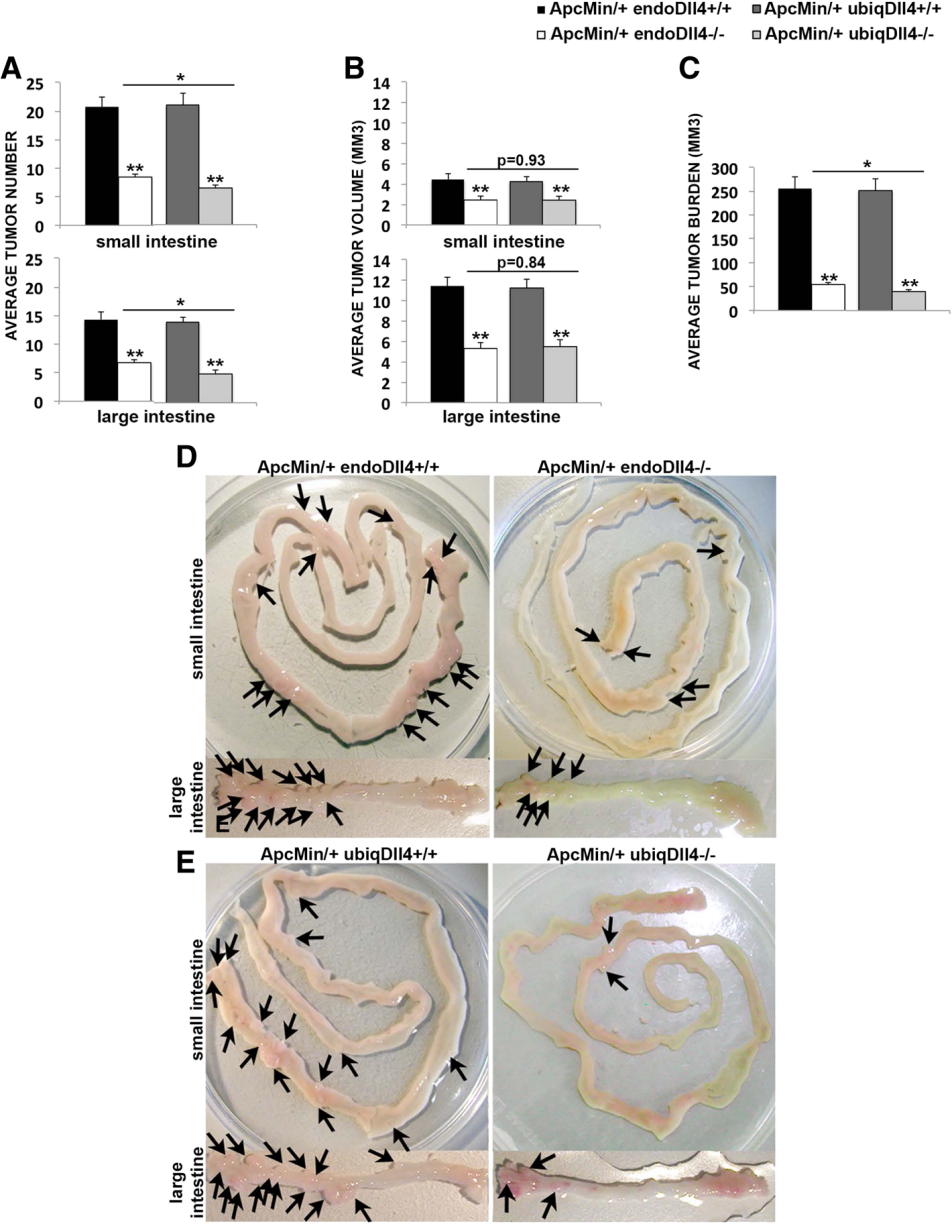
Endothelial-specific and ubiquitous Dll4 loss-of-function inhibits the *Apc* ^*Min/*+^ small and large intestinal tumor development. **a**-**b** *Graphic bars* represent the average ± SEM tumor number (**a**) and volume (**b**) (mm^3^) in the small and large intestine of induced *Apc* ^*Min/*+^ endo*Dll4* ^*-/-*^ and *Apc* ^*Min/*+^ ubiq*Dll4* ^*-/-*^ mice versus their controls (*Apc* ^*Min/*+^ endo*Dll4* ^+*/*+^ and *Apc* ^*Min/*+^ ubiq*Dll4* ^+*/*+^) at 18 weeks of age. **c** Graphic bars represent the average ± SEM tumor burden (mm^3^) in the whole intestine of the animals described above. One experiment with *n* = 12 per group. *P < 0.05; **P < 0.01. **d**-**e** Photographs of the small and large intestines (tumors indicated by *arrows*) collected from the endothelial-specific mutants (**d**) and from the ubiquitous mutants (**e**) versus the respective controls

Notch signaling activation promotes intestinal tumorigenesis mediated by Apc loss of function [13]. In the present study, Dll4 and Jagged1 ligands appear to be the Notch pathway components with greater expression in the small and large *Apc* ^*Min/*+^ adenomas (Figs. 1c and 2c). Previous authors found that the inhibition of Jagged1-mediated Notch signaling is sufficient to reduce the size of the *Apc* ^*Min/*+^ intestinal tumors [39]. However, Dll4/Notch signaling blockade was never evaluated in the *Apc* ^*Min/*+^ model of CRC. It was previously shown that Dll4/Notch signaling is important to maintain the normal gut homeostasis [6] and therefore may also regulate the process of intestinal tumorigenesis. Studies using a xenografted model of CRC suggested that Dll4, besides promoting a dysfunctional vasculature, could have an additional role maintaining the proliferative cancer stem cells [29]. However, this was never studied in premalignant lesions, the initiating event of CRC. We found that in the *Apc* ^*Min/*+^ small and large intestine, Dll4 is strongly expressed in the tumor epithelium, including in the goblet and Paneth cell lineages (Figs. 1c, 2c, and Additional file 4: Figure S1A-B), and it is also present in the tumor endothelium (Additional file 4: Figure S1C-D). In addition, it is present near the Lgr5 positive stem cells in the normal gut and in the intestinal *Apc* ^*Min/*+^ adenomas (Additional file 4: Figure S1E-H). Therefore, we decided to elucidate if Dll4 inhibition could be effective in blocking *Apc* ^*Min/*+^ tumor initiation and development through angiogenic and/or non-angiogenic mechanisms. To that end, *Apc* ^*Min/*+^ mice with endothelial-specific (endo*Dll4* ^*-/-*^) and ubiquitous (ubiq*Dll4* ^*-/-*^) *Dll4* loss-of-function were analysed. At 18 weeks of age both Dll4 mutants had fewer and smaller tumors than the controls (Fig. 4a-e). Endothelial-specific and ubiquitous Dll4 deletion were more effective reducing tumor number than individual tumor growth, both in the small and large intestine (Fig. 4a-b). Intestinal tumorigenesis, but not the size of the tumors, was more affected in the ubiq*Dll4* ^*-/-*^ than in the endo*Dll4* ^*-/-*^ mutants (Fig. 4a-b). Therefore, the overall intestinal tumor burden of the ubiq*Dll4* ^*-/-*^ mice was reduced 6.4-fold, while in the endo*Dll4* ^*-/-*^ mice it was reduced 4.7-fold (Fig. 4c).

In the endo*Dll4* ^*-/-*^ tumors, the efficacy of the endothelial-specific *Dll4* deletion was evaluated through the expression level of the Notch target gene *Hey2* that was found to be decreased by 3.9-fold relatively to the controls. For the ubiq*Dll4* ^*-/-*^ mutants we measured *Dll4* expression, which was reduced 3.4-fold relatively to the controls (Additional file 5: Figure S2).

### Endothelial-specific and ubiquitous Dll4 deletion have similar negative impact in the tumor vasculature, promoting hypoxia and apoptosis in the *Apc*^*Min/*+^ tumors

**Fig. 5:**
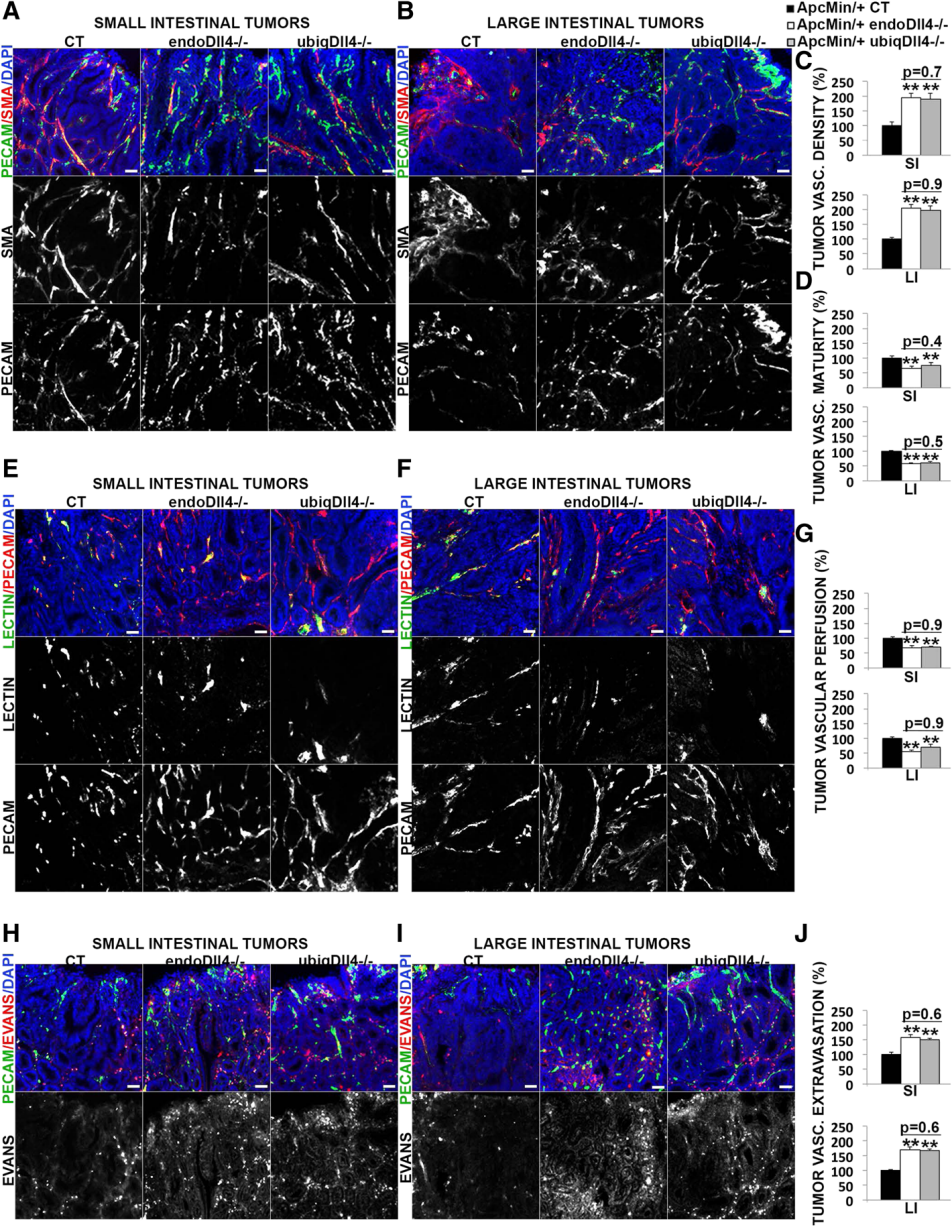
Endothelial-specific and ubiquitous Dll4 deregulation affects similarly the *Apc* ^*Min/*+^ tumor angiogenesis. **a**, **b**, **e**, **f**, **h**, **i** Immunofluorescence stainings of 20 μm small (**a**, **e**, **h**) and large (**b**, **f**, **i**) intestinal tumor cryosections from *Apc* ^*Min/*+^ endo*Dll4* ^*-/-*^, *Apc* ^*Min/*+^ubiq*Dll4* ^*-/-*^ mice and controls (CT) at 18 weeks of age. Scale bars = 100 μm. Representative images of staining density for PECAM-1 (in *green*) and α-SMA (in *red*) (**a**, **b**), for lectin (in *green*) and PECAM-1 (in *red*) (**e**, **f**), and for PECAM-1 (in *green*) and Evans blue (in *red*) (**h**, **i**). The nuclei were counterstained with DAPI (in *blue*). **c**, **d**, **g**, **j** Graphic bars represent the relative (%) ± SEM small (SI) and large (LI) intestinal tumor vascular density (**c**), maturity (**d**), perfusion (**g**) and extravasation (**j**) in the animals described above. One experiment with *n* = 6 per group and 6 fields per animal. *P < 0.05; **P < 0.01

Given the critical role of Dll4 on tumor angiogenesis [24–27], we analysed the tumor vasculature, hypoxia and apoptosis in the endothelial and ubiquitous mutants. The vascular density of the tumors from the small and large intestine was increased similarly in the endo*Dll4* ^*-/-*^ and ubiq*Dll4* ^*-/-*^ mice (Fig. 5a-c). The tumor vascular maturity was evaluated as the density of smooth muscle cells surrounding the wall of the tumor vessels. In the endo*Dll4* ^*-/-*^ and ubiq*Dll4* ^*-/-*^ mice there was a similar reduction of these smooth muscle cells in the blood vessels of both small and large intestinal tumors (Fig. 5a-b and d). The functionality of the tumoral vasculature was evaluated by measuring vessel perfusion and extravasation. In the endo*Dll4* ^*-/-*^ and ubiq*Dll4* ^*-/-*^ mice the tumors presented a similar reduction of the vascular perfusion (Fig. 5e-g) and an equivalent increase of the vascular extravasation in both small and large intestine (Fig. 5h-i).

**Fig. 6:**
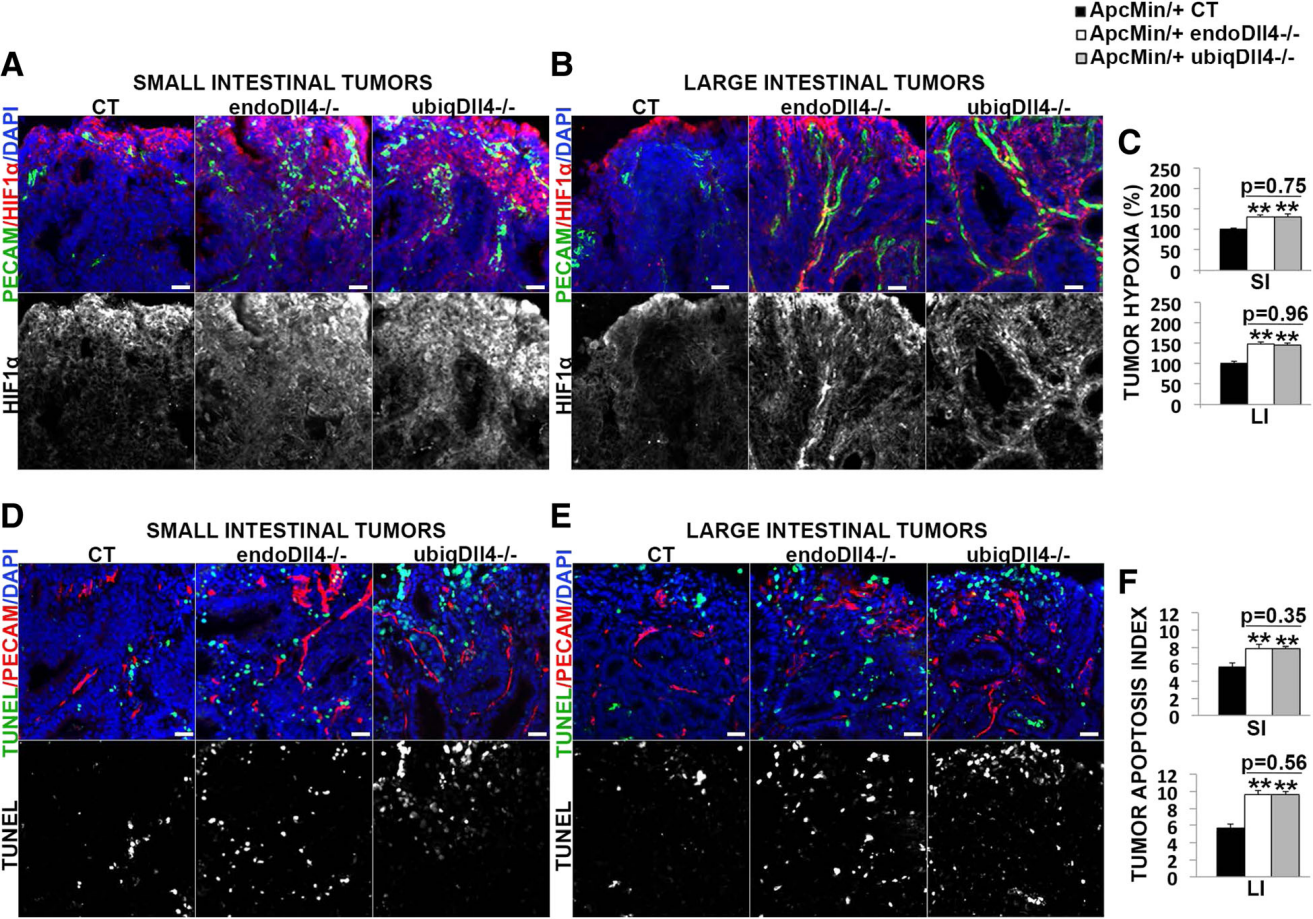
Endothelial-specific and ubiquitous Dll4 deregulation promotes equally hypoxia and apoptosis in *Apc* ^*Min/*+^ tumors. **a**, **b**, **d**, **e** Immunofluorescence stainings of 20 μm (**a**, **b**) and 10 μm (**d**, **e**) cryosections of small (**a**, **d**) and large (**b**, **e**) intestinal tumors collected from *Apc* ^*Min/*+^ endo*Dll4* ^*-/-*^, *Apc* ^*Min/*+^ ubiq*Dll4* ^*-/-*^ and control (CT) mice at 18 weeks of age. Scale bars = 100 μm. Representative images of staining density for PECAM-1 (in *green*) and HIF1α (in *red*) (**a**, **b**), and for TUNEL (in *green*) and PECAM-1 (in *red*) (**d**, **e**). The nuclei were counterstained with DAPI (in *blue*). **c**, **f** Graphic bars represent the relative (%) ± SEM small (SI) and large intestinal (LI) tumor hypoxia (**c**) and tumor apoptotic index (**f**) in the mice described above. One experiment with *n* = 6 per group and 6 fields per animal. *P < 0.05; **P < 0.01

To evaluate tumor hypoxia, tumoral HIF1α density was measured. Both endo*Dll4* ^*-/-*^ and ubiq*Dll4* ^*-/-*^ tumors had an equal increase of the hypoxia level in the small and large intestine (Fig. 6a-c).

The apoptotic index, measured using the TUNEL assay, was similarly increased in the tumors of the small and large intestine in endo*Dll4* ^*-/-*^ and ubiq*Dll4* ^*-/-*^ mutants (Fig. 6d-f).

### Ubiquitous deletion of Dll4 has a significantly stronger effect than endothelial-specific Dll4 deletion in the inhibition of *Apc*^*Min/*+^ tumor proliferation and neoplastic transformation

**Fig. 7:**
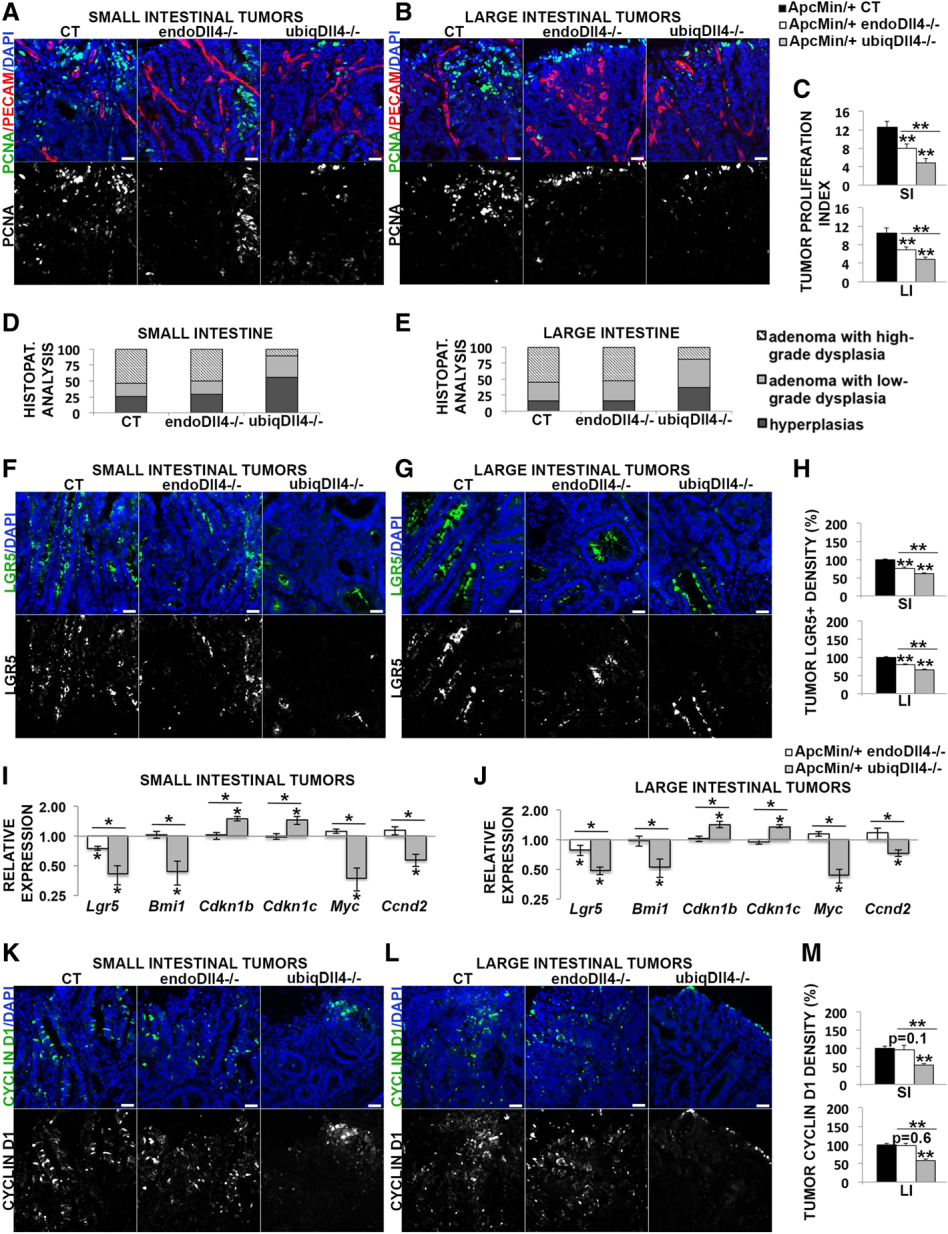
Ubiquitous deletion of Dll4 has a greater inhibitory effect in the *Apc* ^*Min/*+^ tumorigenesis and neoplastic transformation than the endothelial-specific deletion. **a**, **b**, **f**, **g**, **k**, **l** Immunofluorescence stainings of small (**a**, **f**, **k**) and large (**b**, **g**, **l**) intestinal tumor cryosections (10 μm) from *Apc* ^*Min/*+^ endo*Dll4* ^*-/-*^ and ubiq*Dll4* ^*-/-*^ mice versus the controls (CT) at 18 weeks of age. Representative images of staining density for PCNA (in *green*) and PECAM-1 (in *red*) (**a**, **b**), for Lgr5 (in *green*) (**f**, **g**), and for Cyclin D1 (in *green*) (**k**, **l**). The nuclei were counterstained with DAPI (in *blue*). Scale bars = 100 μm. **c**, **h**, **m** Graphic bars represent the small (SI) and large intestinal (LI) tumor proliferation index (**c**), and the relative tumor Lgr5 (**h**) and Cyclin D1 (**m**) density (%) ± SEM in the animals described above. One experiment with *n* = 6 per group and 6 fields per animal. **d**, **e** Graphic bars represent the proportion (%) of hyperplasias and adenomas with low and high-grade dysplasia obtained in the histopathological analysis (H&E) of the macroscopic small (**d**) and large (**e**) intestinal lesions from *Apc* ^*Min/*+^ endo*Dll4* ^*-/-*^, ubiq*Dll4* ^*-/-*^ and control (CT) mice at 18 weeks of age. One experiment with *n* = 12 per group. **i**, **j** RT-PCR analysis of *Lgr5*, *Bmi1*, *Cdkn1b*, *Cdkn1c*, *Myc*, *Ccnd2* relative expression in the *Apc* ^*Min/*+^ endo*Dll4* ^*-/-*^ and ubiq*Dll4* ^*-/-*^ small (**i**) and large (**j**) intestinal tumor samples from mice at 18 weeks of age. One experiment with *n* = 3 per group. *P < 0.05; **P < 0.01

In addition to its angiogenic effect, Dll4/Notch seems to regulate the intestinal cancer cells through other mechanisms [28]. Therefore, we analysed the effect of endothelial-specific versus ubiquitous *Dll4* deletion on tumor proliferation. We observed that the small and large intestinal tumor cell proliferation was reduced in the endo*Dll4* ^*-/-*^ and mainly in the ubiq*Dll4* ^*-/-*^ mice (Fig. 7a-c). The ubiq*Dll4* ^*-/-*^ mice exhibited a statistically significant stronger reduction of tumor proliferation than the endo*Dll4* ^*-/-*^ mice both in the small and large intestine (Fig. 7a-c).

Additionally, we analysed if Dll4 deregulation was affecting the *Apc* ^*Min/*+^ neoplastic transformation. The samples were histopathologically classified in hyperplasia, adenoma with low-grade dysplasia and adenoma with high-grade dysplasia (Additional file 6: Figure S3). No adenocarcinoma lesions were detected. The *Apc* ^*Min/*+^ small and large intestinal neoplastic transformation seemed to be only delayed in the ubiq*Dll4* ^*-/-*^ mice, as they presented substantially more lesions of hyperplasia and less adenomas with high-grade dysplasia comparative to the controls in the large and, mainly, in the small intestine (Fig. 7d-e). Thus, in the controls and endo*Dll4* ^*-/-*^ mice the majority of the small and large intestinal lesions were adenomas with high-grade dysplasia, while in the ubiq*Dll4* ^*-/-*^ mice most of the lesions were hyperplasias and adenomas with low-grade dysplasia in the small and large intestine, respectively (Fig. 7d-e).

### Ubiquitous deletion of *Dll4* has a stronger inhibitory effect than the endothelial-specific blockade on *Apc*^*Min/*+^ tumor stem cell maintenance independently of β-catenin activation

Recent studies showed that the mitotically active Lgr5-positive and the quiescent Bmi1- positive stem cells are responsible for intestinal tumorigenesis [15–17] and that this is regulated by Notch signaling [20, 21]. Therefore, given the observed reduction in the *Apc* ^*Min/*+^ small and large intestinal tumor number in the *Dll4* knock-out mice compared with the controls, we decided to analyse if Dll4 reduction was affecting the Lgr5+ and/or Bmi1+ stem cell expression in the tumors. Compared with the controls, Lgr5 protein and gene expression was reduced in the small and large intestinal tumors from both mutants, but mostly in those from ubiq*Dll4* ^*-/-*^ mice (Fig. 7f-j). Additionally, as previously shown by Pellegrinet et al., we did not observe differences in the density of Lgr5-positive stem cells in the adjacent normal intestine of the mutants [6] (data not shown). In the case of the Bmi1 stem cell marker, its expression was only reduced in the ubiq*Dll4* ^*-/-*^ mice, in both small and large intestinal tumors (Fig. 7i-j). The differences between the two types of *Dll4* mutants in the expression of Lgr5 and Bmi1 markers in the small and large intestinal tumors were statistically significant (Fig. 7f-j).

Previous reports indicated that Notch signaling seems to maintain the intestinal stem cells by transcriptionally repressing the cyclin-dependent kinase inhibitors 1B (*Cdkn1b*) and 1C (*Cdkn1c*) [7]. Accordingly, we observed an increase of *Cdkn1b* and *Cdkn1c* gene expression only in the ubiq*Dll4* ^*-/-*^ small and large intestinal tumors relatively to the controls and to the endo*Dll4* ^*-/-*^ mice (Fig. 7i-j).

Myc and cyclin D1 and D2 are important regulators of intestinal stem cells and are implicated in tumor initiation and progression [40–42]. We observed they were all downregulated only in the ubiq*Dll4* ^*-/-*^ small and large intestinal tumors, mostly in the first region, relatively to the controls and to the endo*Dll4* ^*-/-*^ mice (Fig. 7i-m).

Nonetheless a more thorough analysis should be addressed to confirm our results at the protein level.

Given the central role of the Wnt signaling in *Apc* ^*Min/*+^ tumorigenesis (Sansom, 2004), we measured the tumor density of the Wnt pathway-derived non-phosphorylated (active) β-catenin to understand if Dll4 ubiquitous and/or endothelial-specific inhibition was affecting this pathway. We did not observe statistically significant differences in either of the mutants (Additional file 7: Figure S4A-B).

### Ubiquitous, but not endothelial-specific deletion of Dll4, promotes epithelial differentiation and secretory lineage commitment in the *Apc*^*Min/*+^ tumors

It has been reported that Notch signaling is required for repression of secretory cell differentiation in colon cancer [12]. Blocking this pathway by removal of its transcription factor *RBP-J*_*k*_ or by treatment with gamma-secretase inhibitors, causes a complete conversion of normal and tumoral intestinal proliferative cells into post-mitotic goblet cells [8, 39]. This raises the possibility that the observed reduction of tumoral stem cells might be related to differentiation towards the secretory cell lineages. Therefore we evaluated the density of the epithelial differentiation marker E-cadherin [43] and determined the density of Paneth cells in the tumors, by measuring the level of lysozyme (which is produced by these cells) and the proportion of goblet cells. Paneth cells are normally present only in the small intestine, but previous reports showed Paneth cell metaplasia in large intestinal adenomas [44].

**Fig. 8:**
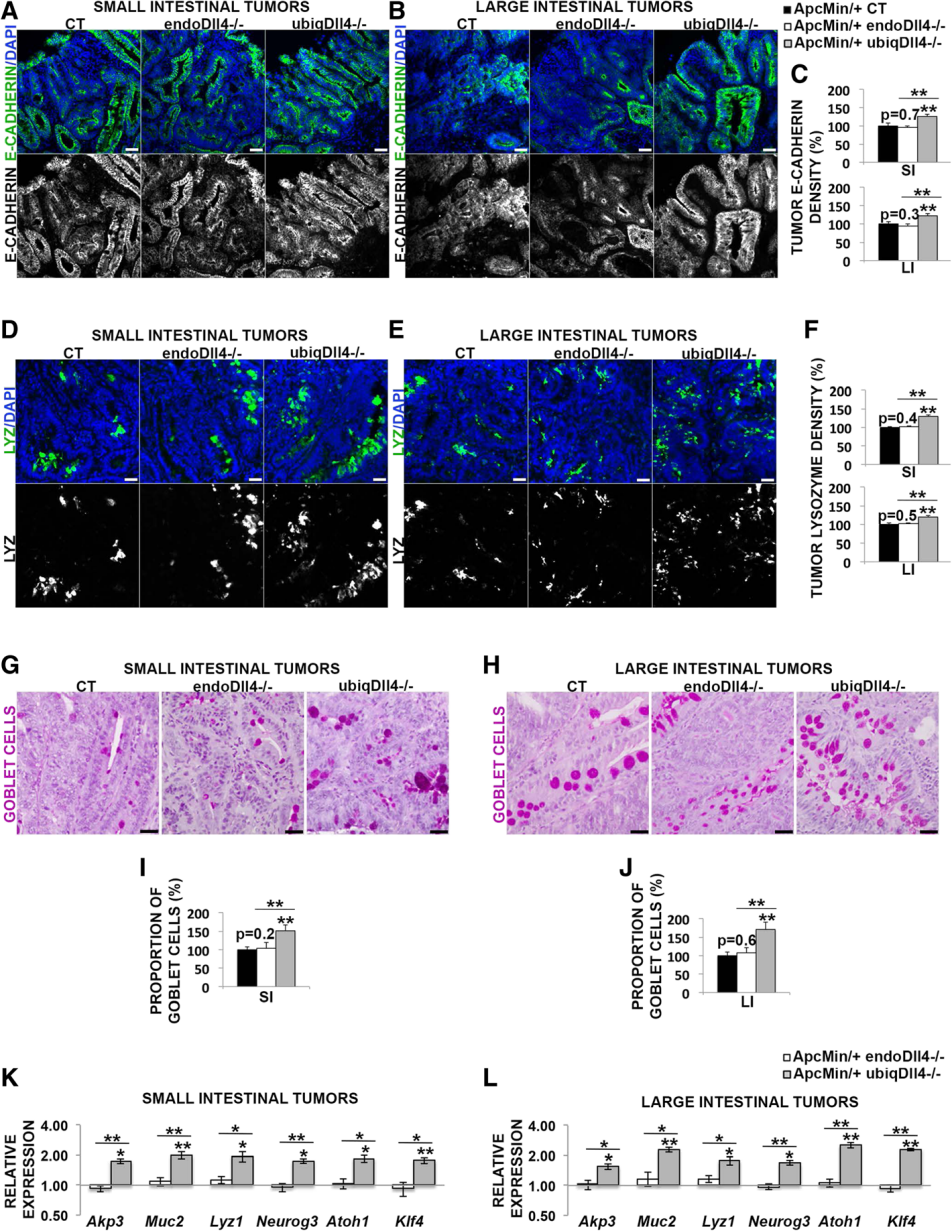
Ubiquitous, but not endothelial-specific, loss of Dll4 promotes intestinal differentiation towards the secretory lineages. **a**, **b**, **d**, **e** Immunofluorescence stainings of small (**a**, **d**) and large (**b**, **e**) intestinal tumor cryosections (10 μm) from *Apc* ^*Min/*+^ endo*Dll4* ^*-/-*^ and ubiq*Dll4* ^*-/-*^ mice versus controls (CT) at 18 weeks of age. Representative images of staining density for E-cadherin (in *green*) (**a**, **b**) and lysozyme (in *green*) (**d**, **e**). Nuclei were counterstained with DAPI (in *blue*). Scale bars = 100 μm. **c**, **f** Graphic bars represent the tumor relative (%) ± SEM density of E-cadherin (**c**) and lysozyme (**f**) in the small (SI) and large (LI) intestinal tumors from the animals described above. One experiment with *n* = 6 per group and 6 fields per animal. **g**, **h** PAS staining (of goblet cells) of paraffin-embedded small (**g**) and large (**h**) intestinal tumor sections (4 μm) from *Apc* ^*Min/*+^ endo*Dll4* ^*-/-*^ and ubiq*Dll4* ^*-/-*^ mice versus controls (CT) at 18 weeks of age. Scale bars = 100 μm. **i**, **j** Graphic bars represent the relative proportion (%) ± SEM of goblet cells in the small (**i**) and large (**j**) intestinal tumor epithelium from the animals described above. One experiment with *n* = 6 per group and 6 fields per animal. **k**, **l** RT-PCR analysis of *Akp3*, *Muc2*, *Lyz*, *Neurog3*, *Atoh1*, *Klf4* relative expression in the *Apc* ^*Min/*+^ endo*Dll4* ^*-/-*^ and *Apc* ^*Min/*+^ ubiq*Dll4* ^*-/-*^ small (**k**) and large (**l**) intestinal tumor samples. One experiment with *n* = 3 per group. *P < 0.05; **P < 0.01

Relatively to the controls, we found a moderate increase of tumor epithelial differentiation in the ubiq*Dll4* ^*-/-*^, but not in the endo*Dll4* ^*-/-*^, small and large intestine (Fig. 8a-c). Additionally, the density of Paneth cells and mainly the proportion of goblet cells were increased only in the ubiq*Dll4* ^*-/-*^ small and large intestinal tumors (Fig. 8d-j). The differences between the two Dll4 mutants in the level of epithelial differentiation and specifically in the density of Paneth and goblet cells were statistically significant (Fig. 8a-j).

We also measured the relative transcription in the tumors of *Akp3*, *Muc2*, *Lyz1* and *Neurog3* markers for the enterocyte, goblet, Paneth and neuroendocrine cell populations, respectively, and also of the promoters of secretory lineage commitment *Atoh1* and *Klf4* [10, 45]. Compared with the controls, all the lineage markers evaluated were significantly increased in the ubiq*Dll4* ^*-/-*^ tumors, mostly *Muc2*, and *Lyz1* in the small intestine and *Muc2*, *Atoh1 and Klf4* in the large intestine (Fig. 8k-l). No significant differences were observed in the endo*Dll4* ^*-/-*^ small and large intestinal tumors relatively to the controls (Fig. 8k-l). Nevertheless, further analysis should be considered to verify if there is a correlation between all the transcriptional data at the protein level.

## Discussion

Activation of Notch pathway seems to promote intestinal tumorigenesis induced by *Apc* loss [13] and Dll4 is one of the Notch signaling pathway components found to be upregulated in these tumors [46]. Reports have shown that Dll4 inhibition delays the tumor growth by deregulating the tumor angiogenic process [24–27], but in CRC anti-DLL4 therapy may also reduce the cancer stem cell frequency [29]. Despite the advances in the understanding of Dll4/Notch signaling in cancer, most of the previous reports were focused on role of Dll4 in the tumor angiogenic process and further studies are still needed to unveil all the mechanisms by which Dll4 affects the tumor initiation and development in the gut.

Reports have shown that all Notch receptors, ligands and some of the Notch target genes are expressed in the normal gut [8, 37, 38]. However, in the *Apc* ^*Min/*+^ intestinal tumors their expression has been poorly described. A study indicated that expression of Notch receptors and ligands closely follows the expression in the normal crypts, while Hes1 expression was observed uniformly in the adenomas [8]. Other report showed that Jagged1 is overexpressed in the tumor tissue with concomitant Notch1 and 2 activation [39]. In the present work we analysed the protein expression pattern of most Notch pathway members in the *Apc* ^*Min/*+^ intestinal tumors compared with the normal WT gut. Regarding the previous gene expression analysis of Notch members in the normal gut [37, 38], we observed some differences in our analysis. These included the presence of Notch2 in the bottom of the large intestinal crypts, of Notch3 and 4 in the small and large intestinal epithelium and Hes1 not only in the small intestinal crypts [37], but diffusely expressed in the small and large intestine.

Our expression analysis in the *Apc* ^*Min/*+^ small and large intestinal adenomas confirmed that the Notch pathway is present and activated in intestinal adenomas harbouring *Apc* mutations [8, 13, 39, 46]. Dll4 and Jagged1 were more expressed in these tumors than the other members of this pathway. Comparing the adenomas with the normal WT gut we found that Dll1 and Dll3 lose their expression in the large intestine. We observed a different expression pattern of Dll4, all Notch receptors (with regional variation) and Hes5 in the tumor epithelium. The same Notch members and Hes1, instead of Hes5, seemed upregulated in the adenomas (Notch3 only in the large intestine). Thus our expression analysis indicates that in the *Apc* ^*Min/*+^ small and large intestinal adenomas, Dll4 is the most upregulated ligand and is present both in the tumor epithelium and endothelium.

In the normal gut Dll4 acts redundantly with Dll1 mediating the Notch signaling regulation of the intestinal stem cells proliferation and their commitment towards de secretory cell fate [6]. We found that Dll4 is expressed near the Lgr5+ stem cells also in the intestinal tumors, therefore indicating a possible role of this ligand in the maintenance of also the tumor stem cells. These stem cells are believed to be responsible for tumor initiation and progression [47, 48] and depend on proper angiogenesis to function and survive [22]. Therefore we intended to elucidate if Dll4 also regulates the fate of tumor stem cells beside its angiogenic effect in a spontaneous model of CRC, the *Apc* ^*Min/*+^ mouse. To address this question we compared ubiquitous with endothelial-specific Dll4 loss-of-function mouse mutants. Our results highlighted the importance of Dll4 angiogenic and epithelial effect during intestinal *Apc* ^*Min/*+^ tumor initiation and development rather than in maintaining the normal gut homeostasis. Pellegrinet el al. reported that in the normal gut, *Dll4* intestinal epithelial-specific inhibition alone is not sufficient to promote a phenotype due to redundant Dll1-mediated Notch signaling [6]. This lack of intestinal effect after *Dll4* inhibition can also be related to the fact that in the normal gut simultaneous inhibition of *Notch1* and *2* is necessary to result in complete conversion of the crypt progenitors into postmitotic goblet cells [7] and it is not known whether Dll4 can activate both receptors in the gut. Nevertheless, our results show that Dll4 seems at least partially responsible for the known effects of Notch activation during intestinal tumorigenesis, as Dll4 ubiquitous deletion led to a similar, but less pronounced, epithelial phenotype as the pan-Notch/*gamma*-secretase inhibition in the *Apc* ^*Min/*+^ tumors [8]. However, as the alterations were moderated with no macroscopic repercussions (no observed increase of mucus secretion) in the ubiquitous Dll4 mutants’ gut, Dll1 may partially compensate the lack of Dll4 in this setting and/or Dll4 may not activate both Notch1 and 2 receptors. In addition, as we only analysed the Lgr5 and Bmi1 positive stem cell populations, it is not certain if this pathway can affect similarly all the stem cells present in the intestinal tumors.

We found that ubiquitous and endothelial-specific Dll4 blockade led to a similar phenotype in the small and large intestine, but a stronger effect on tumor initiation was observed in the small intestine and a greater impact on tumor growth was seen in the large intestine.

By comparing the ubiquitous mutants with the endothelial-specific knockouts we found that the observed epithelial phenotype is probably caused by the deregulation of the tumor angiogenesis but also by other important mechanisms. Both ubiquitous and endothelial-specific mutants had an equivalent angiogenic phenotype, with equally increased hypoxia and apoptosis leading to similar reduction of the tumor volume. Therefore, Dll4 deletion inhibited the intestinal tumor growth by inducing a dysfunctional and immature angiogenesis that led to hypoxia and therefore apoptosis as previously reported in other tumor models [24, 26, 27, 49].

The multiplicity of tumors was also reduced in the mutants relatively to their controls and this effect was more pronounced in the ubiquitous than in the endothelial-specific mutants, associated to a stronger reduction of tumor cell proliferation and tumor stem cell density in the first mutants. The stronger inhibitory effect on tumor cell proliferation through Dll4 ubiquitous deletion may have therefore prevented the accumulation of more mutations that lead to tumor initiation, promote the transition of microadenomas to macroadenomas and favor the neoplastic transformation. Therefore in the intestinal adenomas, Dll4 seems to promote proliferation and maintain the stem cells through angiogenic, but also non-angiogenic related mechanisms. Indeed we observed decreased expression of Myc, cyclin D1 and D2, independently of β-catenin activation, only in the ubiq*Dll4* ^*-/-*^ tumors.

The Wnt signaling has been considered a crucial player in the initiation of CRC associated to inactive mutations in the *APC* gene [3]. Nuclear accumulation of β-catenin promotes neoplastic conversion by triggering the cell cycle-regulators Cyclin D1 and D2 and Myc and, consequently, uncontrolled cell proliferation contributing to tumor progression [40, 50, 51]. Notch signaling seems to cooperate with Wnt signaling to trigger intestinal tumorigenesis, as activation of Notch in *Apc* mutant mice led to a significant increase in the number of adenomas developed [13]. Additionally, a previous study indicated that Jagged1 was the link between Wnt and Notch pathways in the *Apc* ^*Min/*+^ tumorigenesis, where β-catenin seems to transcriptionally activate Jagged1 [39]. However, it has been shown that the Mastermind-like 1 co-activator of Notch pathway can bind to the promoters of Cyclin D1 and Myc in colon cancer cell lines [52] and these molecules are activated directly by Notch1 in other types of cancer [53–57] and possibly by Cyclin D1 in CRC [58]. It has been also demonstrated that Cyclin D2 and Myc are also induced by Notch1 to promote stem cell renewal in another setting [59]. Additionally, the overexpression of Dll4 in a leukemia cell line led to increased protein expression of Myc [60]. Therefore, during *Apc* ^*Min/*+^ tumorigenesis Dll4/Notch signaling may directly upregulate the expression of Cyclin D1 and D2 and Myc. We observed that Dll4 deletion reduced tumorigenesis without affecting β-catenin nuclear accumulation and thus Wnt activation. Therefore, Dll4/Notch activation may promote intestinal tumorigenesis by angiogenic and non-angiogenic mediated mechanisms in a β-catenin independent manner. The non-angiogenic related regulation may include a synergistic effect of Dll4/Notch with Wnt signaling to promote tumorigenesis by increasing the transcription of important Wnt target genes.

In addition, Dll4 ubiquitous inhibition upregulated the zinc finger-containing transcription factor KLF4 in the *Apc* ^*Min/*+^ tumors. KLF4 is a cell proliferation inhibitor and can act as a tumor suppressor, being normally downregulated in *Apc* ^*Min/*+^ tumors and in early stages of human CRC [61]. The loss of one of its alleles increases *Apc* ^*Min/*+^ tumorigenesis, possibly by derepressing β-catenin mediated gene expression [62]. A previous work indicated that Notch signaling supresses KLF4 expression in intestinal tumors and colorectal cancer cells [62]. Our results indicate that Dll4 seems to be the ligand responsible for this Notch-mediated phenotype. Therefore, Dll4/Notch may promote carcinogenesis by upregulating the transcription of Wnt target genes through KLF4 downregulation in the *Apc* mutated tumors. Previous work indicated that Hes1 downregulation by Notch inhibition derepresses Atoh1, which seems to induce KLF4 upregulation to promote goblet cell differentiation in a redundant manner [11, 63]. However, it seems that Hes1 may act both upstream and downstream of Atoh1 to negatively regulate KLF4 [11]. We found that in the ubiquitous, but not in the endothelial-specific, Dll4 knockouts, the reduction of Lgr5 and Bmi1 positive tumor stem cell density was accompanied with increased tumor epithelium differentiation with a moderate deviation towards the secretory lineages, probably due to the observed *Atoh1* and *Klf4* overexpression by *Hes1* downregulation as it occurs when Notch signalling is inhibited [8, 63]. This indicates that besides the effect on angiogenesis, Dll4/Notch signaling seems to have an additional role maintaining the tumor stem cells undifferentiated.

Additionally, Dll4/Notch ubiquitous inhibition promoted the transcription of the cell cycle regulators cyclin-dependent kinase (CDK) inhibitors *Cdkn1b* and *Cdkn1c* in the *Apc* ^*Min/*+^ tumors. A previous report showed that inactivation of Notch1 and 2 in the normal gut is associated with derepression of these CDK inhibitors [7]. This phenotype was completely abrogated in the absence of Atoh1 [7, 64], a molecule that is also considered to act as a tumor suppressor in CRC [65]. Therefore, Dll4/Notch inhibition may also negatively affect the tumor stem cell populations through Atoh1 derepression-mediated upregulation of the CDK inhibitors *Cdkn1b* and *Cdkn1c*.

## Conclusions

In summary, we show that Dll4 seems to be the ligand responsible, at least partially, for the previously reported Notch effects during intestinal tumor development. Dll4/Notch deletion seems to inhibit the initiation and development of intestinal tumorigenesis through angiogenic and non-angiogenic related mechanisms independently of β-catenin activation and without affecting the normal gut. The non-angiogenic associated effects mediated by this pathway blockade may include the inhibition of tumor cell proliferation, the neoplastic transformation, the maintenance of the tumor stem cells and the promotion of epithelial differentiation predominantly towards the secretory cell lineages.

Thus, despite the need for further studies, Dll4/Notch blockade appears to be a good candidate strategy to prevent CRC in patients predisposed to this disease and should also be considered in the treatment of early stages of CRC.

## Additional files

Additional file 1: ARRIVE checklist. (PDF 617 kb)
Additional file 2: Table S1. Primer pair sequences list used in RT-PCR reactions. (XLSX 53 kb)
Additional file 3: Table S2. Summary of sites of Notch pathway protein expression in the normal small and large intestine. Dll1, 3 and 4 Jagged1 and 2, Notch1-4, Hes1 and 5 protein expression pattern (indicated by an x) in the small intestine crypts and villus epithelium, in the bottom and top of the large intestine crypts and in the *lamina propria* of the normal WT intestine of C57BL/6 mice at 18 weeks of age. One experiment with *n* = 2 per group, 3 slides per mouse, and 2 sections per slide. (XLSX 38 kb)
Additional file 4: Figure S1. Dll4 is expressed in tumoral Paneth cells and endothelium, and near tumoral and normal Lgr5+ cells. (A, B) Representative images of the immunofluorescence co-staining of Dll4 (in red) with lysozyme (produced by Paneth cells and stained in green) in the *Apc* ^*Min/*+^ small (A) and large (B) intestinal adenomas at 18 weeks of age. (C, D) Representative images of the immunofluorescence co-staining of Dll4 (in green) with PECAM-1 (in red) in the small (C) and large (D) intestinal adenomas. (E-H) Representative images of the immunofluorescence co-staining of Dll4 (in red) with the Lgr5 stem cell marker (in green) in the normal small (E) and large (F) intestine and in adenomas from the small (G) and large (H) intestine. Nuclei were counterstained with DAPI (in blue). One experiment with *n* = 2 per group and 6 fields per animal. Scale bar = 50 μm. SI, small intestine; LI, large intestine. (PDF 230 kb)
Additional file 5: Figure S2. Confirmation of Dll4 knockout in endothelial-specific and ubiquitous Dll4 *Apc* ^*Min/*+^ mutant tumors. RT-PCR analysis of the Dll4/Notch effector *Hey2* relative expression in the *Apc* ^*Min/*+^ endo*Dll4* ^*-/-*^ intestinal tumors and of *Dll4* relative expression in the *Apc* ^*Min/*+^ ubiq*Dll4* ^*-/-*^ intestinal tumors, both from mice at 18 weeks of age. One experiment with *n* = 3 per group. The expression of *Hey2* and *Dll4* was normalized to *Pecam-1*. **P* < 0.05. (PDF 71 kb)
Additional file 6: Figure S3. Histopathological classification of the *Apc* ^*Min/*+^ small and large intestinal lesions. H&E images of the normal small and large intestine, and of a hyperplasia and adenomas with low and high-grade dysplasia from these regions, the lesions found in the *Apc* ^*Min/*+^ endo*Dll4* ^*-/-*^, *Apc* ^*Min/*+^ ubiq*Dll4* ^*-/-*^ and controls at 18 weeks of age. One experiment with *n* = 12 per group. Scale bars = 100 μm. (PDF 223 kb)
Additional file 7: Figure S4. Dll4 endothelial-specific and ubiquitous deregulation does not affect the intestinal *Apc* ^*Min/*+^ associated β-catenin activation. (A, B) Representative images of the immunofluorescence staining density for non-phosphorylated (active) β-catenin (in green) of the small (A) and large (B) intestine tumor cryosections (10 μm) from *Apc* ^*Min/*+^ endo*Dll4* ^*-/-*^ and *Apc* ^*Min/*+^ ubiq*Dll4* ^*-/-*^ mice versus controls (CT) at 18 weeks of age. The nuclei were counterstained with DAPI (in blue). Scale bars = 100 μm. (C) Graphic bars represent the small and large intestinal relative tumor non-phosphorylated (active) β-catenin density ± SEM in the animals described above. One experiment with *n* = 6 per group and 6 fields per animal. (PDF 309 kb)

## Abbreviations

CDK: Cyclin-dependent kinase
CRC: Colorectal cancer
DAPI: 4′, 6-diamidino-2-phenylindole dihydrochloride hydrate
Dll4: Delta *like* 4
endo*Dll4*^*-/-*^: Endothelial-specific inducible *Dll4* loss-of-function
HIF1α: Hypoxia-inducible factor 1-alpha
PCNA: Proliferating cell nuclear antigen
PECAM-1: Platelet and endothelial cell adhesion molecule 1
ubiq*Dll4*^*-/-*^: Ubiquitous inducible *Dll4* loss-of-function
α-SMA: Alpha smooth muscle actin
