# Erratum to: Delta-like 4/Notch signaling promotes *Apc*^*Min/*+^ tumor initiation through angiogenic and non-angiogenic related mechanisms

**DOI:** 10.1186/s12885-017-3202-z

**Published:** 2017-03-21

**Authors:** Marina Badenes, Alexandre Trindade, Hugo Pissarra, Luís Lopes-da-Costa, António Duarte

**Affiliations:** Centro Interdisciplinar de Investigação em Sanidade Animal (CIISA), University of Lisbon, Lisbon, Portugal

## Erratum

After the publication of this work [1] it was noticed that the reference included in the Funding section was incorrect. It currently reads PEst-OE/AGR/U10276/2014, but it should read UID/CVT/00276/2013.

## References

[1] Badenes M, Trindade A, Pissarra H, Lopes-da-Costa L, Duarte A (2017). Delta-like 4/Notch signaling promotes *Apc*^*Min/*+^ tumor initiation through angiogenic and non-angiogenic related mechanisms. BMC Cancer..

