# Supplementary figures and images for: Delta-like 4/Notch signaling promotes ApcMin/+ tumor initiation through angiogenic and non-angiogenic related mechanisms
Source: BMC Cancer. 2017 Jan 13;17:50. doi: 10.1186/s12885-016-3036-0 (PMC5237288; doi:10.1186/s12885-016-3036-0)

SI ADENOMA

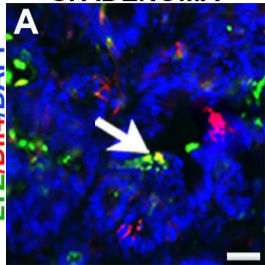

LI ADENOMA

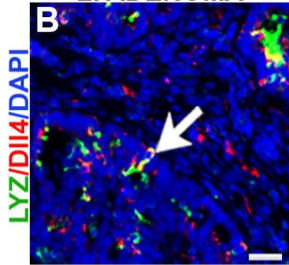

SI ADENOMA

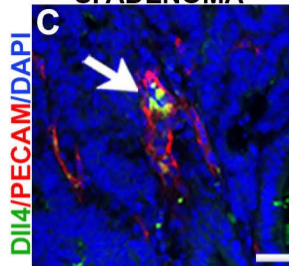

LI ADENOMA

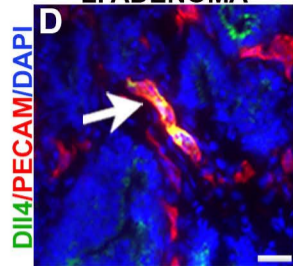

NORMAL SI

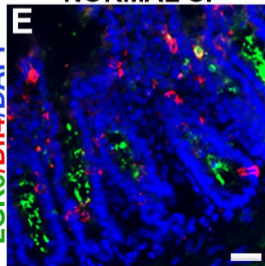

NORMAL LI

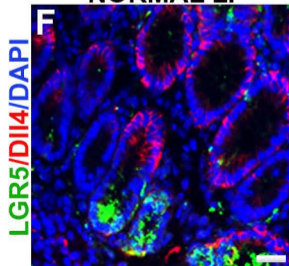

SI ADENOMA

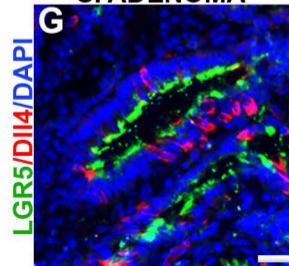

LI ADENOMA

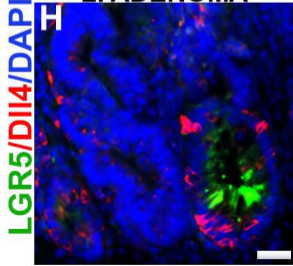

Supplement: Additional file 4: Figure S1. — Dll4 is expressed in tumoral Paneth cells and endothelium, and near tumoral and normal Lgr5+ cells. (A, B) Representative images of the immunofluorescence co-staining of Dll4 (in red) with lysozyme (produced by Paneth cells and stained in green) in the Apc Min/+ small (A) and large (B) intestinal adenomas at 18 weeks of age. (C, D) Representative images of the immunofluorescence co-staining of Dll4 (in green) with PECAM-1 (in red) in the small (C) and large (D) intestinal adenomas. (E-H) Representative images of the immunofluorescence co-staining of Dll4 (in red) with the Lgr5 stem cell marker (in green) in the normal small (E) and large (F) intestine and in adenomas from the small (G) and large (H) intestine. Nuclei were counterstained with DAPI (in blue). One experiment with n = 2 per group and 6 fields per animal. Scale bar = 50 μm. SI, small intestine; LI, large intestine. (PDF 230 kb) [file 12885_2016_3036_MOESM4_ESM.pdf]

RELATIVE  
EXPRESSION

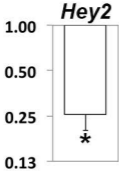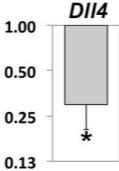

□ ApcMin/+ endoDII4-/-  
■ ApcMin/+ ubiqDII4-/-

Supplement: Additional file 5: Figure S2. — Confirmation of Dll4 knockout in endothelial-specific and ubiquitous Dll4 Apc Min/+ mutant tumors. RT-PCR analysis of the Dll4/Notch effector Hey2 relative expression in the Apc Min/+ endoDll4 -/- intestinal tumors and of Dll4 relative expression in the Apc Min/+ ubiqDll4 -/- intestinal tumors, both from mice at 18 weeks of age. One experiment with n = 3 per group. The expression of Hey2 and Dll4 was normalized to Pecam-1. *P < 0.05. (PDF 71 kb) [file 12885_2016_3036_MOESM5_ESM.pdf]
