# Supplementary material for: Delta-like 4/Notch signaling promotes ApcMin/+ tumor initiation through angiogenic and non-angiogenic related mechanisms
Source: BMC Cancer. 2017 Jan 13;17:50. doi: 10.1186/s12885-016-3036-0 (PMC5237288; doi:10.1186/s12885-016-3036-0)
Supplement: Additional file 6: Figure S3. — Histopathological classification of the Apc Min/+ small and large intestinal lesions. H&E images of the normal small and large intestine, and of a hyperplasia and adenomas with low and high-grade dysplasia from these regions, the lesions found in the Apc Min/+ endoDll4 -/-, Apc Min/+ ubiqDll4 -/- and controls at 18 weeks of age. One experiment with n = 12 per group. Scale bars = 100 μm. (PDF 223 kb) [file 12885_2016_3036_MOESM6_ESM.pdf]

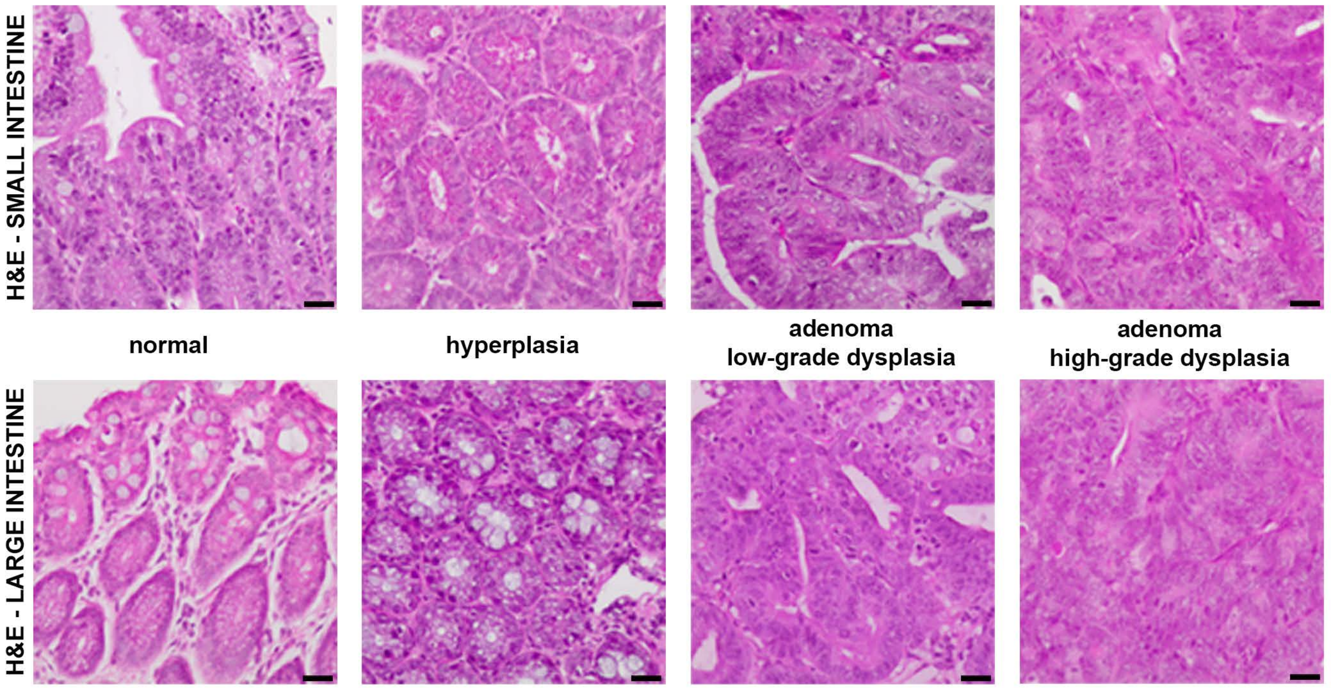
H&E - SMALL INTESTINE
H&E - LARGE INTESTINE
normal
hyperplasia
adenoma
low-grade dysplasia
adenoma
high-grade dysplasia
