# Supplementary material for: Delta-like 4/Notch signaling promotes ApcMin/+ tumor initiation through angiogenic and non-angiogenic related mechanisms
Source: BMC Cancer. 2017 Jan 13;17:50. doi: 10.1186/s12885-016-3036-0 (PMC5237288; doi:10.1186/s12885-016-3036-0)
Supplement: Additional file 7: Figure S4. — Dll4 endothelial-specific and ubiquitous deregulation does not affect the intestinal Apc Min/+ associated β-catenin activation. (A, B) Representative images of the immunofluorescence staining density for non-phosphorylated (active) β-catenin (in green) of the small (A) and large (B) intestine tumor cryosections (10 μm) from Apc Min/+ endoDll4 -/- and Apc Min/+ ubiqDll4 -/- mice versus controls (CT) at 18 weeks of age. The nuclei were counterstained with DAPI (in blue). Scale bars = 100 μm. (C) Graphic bars represent the small and large intestinal relative tumor non-phosphorylated (active) β-catenin density ± SEM in the animals described above. One experiment with n = 6 per group and 6 fields per animal. (PDF 309 kb) [file 12885_2016_3036_MOESM7_ESM.pdf]

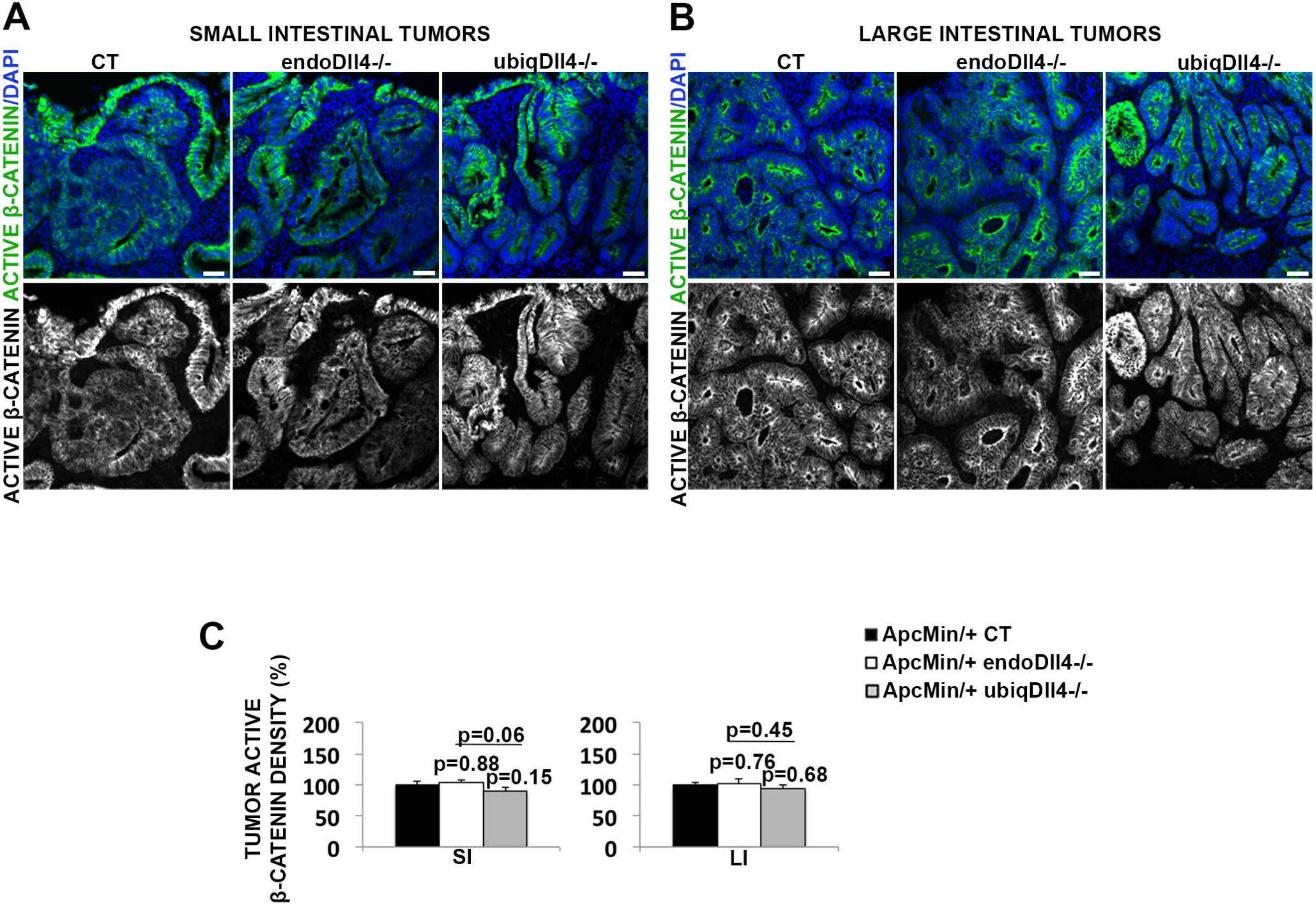
A
SMALL INTESTINAL TUMORS
CT
endoDll4-/-
ubiqDll4-/-
ACTIVE β-CATENIN/DAPI
ACTIVE β-CATENIN
B
LARGE INTESTINAL TUMORS
CT
endoDll4-/-
ubiqDll4-/-
ACTIVE β-CATENIN/DAPI
ACTIVE β-CATENIN
C
ApcMin/+ CT
ApcMin/+ endoDll4-/-
ApcMin/+ ubiqDll4-/-
TUMOR ACTIVE
β-CATENIN DENSITY (%)
200
150
100
50
0
p=0.06
p=0.88
p=0.15
SI
p=0.45
p=0.76
p=0.68
LI
